# Optimizing MALDI-TOF Mass Spectrometry for the Identification of *Bacillus cereus*: The Impact of Sporulation and Cultivation Time

**DOI:** 10.3390/ijms26094355

**Published:** 2025-05-04

**Authors:** Beomyeol Baek, Yoon Ho Park, Ju-Mi Jeon, Hee-Young Shim, Eun-Kyoung Lee, Mi-Jeong Hong, Young-Woo Bae, Joong-Heok An, In-Cheol Shin, Hyun Suk Jung

**Affiliations:** 1 Department of Biochemistry, College of Natural Sciences, Kangwon National University, Chuncheon 24341, Republic of Korea; skyme0311@korea.kr (B.B.); yhpark99@kangwon.ac.kr (Y.H.P.); 2 Infectious Disease Research Division, Gangwon-do Institute of Health and Environment, 386-1, Sinbuk-ro, Sinbuk-eup, Chuncheon-si 24203, Republic of Korea; biotech@korea.kr (J.-M.J.); shimhy@korea.kr (H.-Y.S.); eunice20@korea.kr (E.-K.L.); hmj0818@korea.kr (M.-J.H.); ywbae@kore.kr (Y.-W.B.); aht0209@korea.kr (J.-H.A.); shin6711@korea.kr (I.-C.S.)

**Keywords:** *Bacillus cereus*, MALDI-TOF, VITEK2, microbial identification, bacterial spores

## Abstract

*Bacillus cereus* is a significant foodborne pathogen that presents a critical challenge in food safety due to its ability to form resistant spores and produce various toxins. The potential for severe food poisoning makes rapid and accurate identification of this pathogen essential. Conventional microbiological methods for *B. cereus* identification rely on morphological characteristics and biochemical tests, requiring extensive time and labor. However, even automated biochemical systems like VITEK2, while providing reliable results, still require up to 16 h for analysis and complex sample preparation procedures. MALDI-TOF (matrix-assisted laser desorption/ionization time-of-flight) mass spectrometry utilizes laser-induced ionization of bacterial proteins and subsequent time-of-flight analysis to generate unique mass spectral patterns. This established analytical technique for bacterial identification offers exceptional speed and simplicity through direct protein profiling. In this study, we optimized MALDI-TOF analysis conditions for *B. cereus* identification by examining various cultivation times. Our results demonstrated complete species-level identification accuracy with MALDI-TOF scores ≥ 2.0 with 12-h cultures, matching the reliability of VITEK2 while significantly reducing processing time. The identification rates decreased significantly from 100% at 12 h to 73.3% at 24 h and 50% at 48 h of incubation, correlating directly with increased spore formation. Detailed analysis at 4-h intervals revealed that high identification rates (93.3%) were maintained during 16 h of cultivation before declining significantly. This study establishes MALDI-TOF as a reliable and efficient tool for rapid *B. cereus* identification, representing a significant advancement in food safety diagnostics with potential time savings of more than 50% compared to conventional methods.

## 1. Introduction

*Bacillus cereus* is a significant foodborne pathogen that presents a critical challenge in food safety due to its ability to form resistant spores and produce various toxins [1]. This Gram-positive bacterium causes two distinct types of foodborne illness: the emetic syndrome, characterized by nausea and vomiting, and the diarrheal syndrome, resulting in abdominal pain and diarrhea [2,3]. The public health significance of *B. cereus* is amplified by its production of various toxins, particularly cereulide, a heat-stable emetic toxin [4]. Cereulide remains active even after exposure to high temperatures (e.g., 121 °C for 90 min) and can persist through conventional cooking processes, potentially causing serious emetic symptoms even when bacterial cells are no longer viable [4]. This characteristic poses significant challenges for food safety protocols and highlights the need for effective detection and control strategies, especially considering the growing incidence of foodborne illness and nosocomial outbreaks associated with this species [2,3]. Consequently, *B. cereus* is a critical target for rapid identification methods.

The ability of *B. cereus* to form highly resistant endospores represents both a survival advantage for the bacterium and a significant challenge for detection methods [5]. These spores consist of multiple protective layers that provide remarkable resistance to heat, desiccation, radiation, and chemical agents [6,7]. As vegetative cells transition to spores, their biochemical and structural properties undergo significant alterations, affecting the reliability of conventional identification methods [8]. This spore-forming capacity is especially concerning for vulnerable populations, such as immunocompromised patients, neonates, and critically ill individuals, where *B. cereus* invasive infections can lead to mortality rates of 10–42% [9,10,11]. This significantly exceeds the average mortality rates associated with other common foodborne pathogens such as *Salmonella* spp. (0.5%) or *E. coli* O157 (0.6%) [12].

The public health threat posed by *B. cereus* has been further emphasized in recent reviews, which highlight the growing incidence of foodborne illness and nosocomial outbreaks associated with this species [2,3]. Of particular concern is cereulide, a heat-stable emetic toxin that can persist during cooking and cause serious symptoms, even when bacterial cells are no longer viable [4]. These characteristics make *B. cereus* a critical target for rapid identification methods, particularly in vulnerable populations and institutional settings. Moreover, the findings from this study regarding the influence of sporulation on MALDI-TOF MS identification accuracy may be relevant to other spore-forming genera, such as *Bacillus subtilis*, *Bacillus anthracis*, and *Clostridium* spp. These organisms undergo comparable proteomic shifts during sporulation, as reported in *Bacillus cereus* and *Bacillus subtilis* [8,13,14], suggesting that the optimized cultivation time window proposed here could be broadly applicable across multiple clinically significant spore-forming bacteria and serve as a practical reference for developing rapid identification protocols in food safety and clinical diagnostics.

Traditional microbiological identification of *B. cereus* relies on morphological characteristics and biochemical tests, which are both time-consuming and labor-intensive [15]. Even automated systems such as VITEK2 require pure colonies from initial cultivation (18–24 h), followed by an additional 8–10 h for biochemical analysis, resulting in total identification times of 26–34 h [16]. Although molecular methods such as PCR are widely used for genotypic characterization and toxin gene detection, they were not the primary focus of this study. PCR requires multiple preparatory steps (e.g., DNA extraction, thermal cycling) and can detect DNA from non-viable cells, which may not reflect the actual infection risk in foodborne outbreaks. In contrast, MALDI-TOF MS enables direct identification from viable colonies with minimal preparation, offering clear advantages for rapid diagnostics. While PCR-based toxin gene profiling was performed in parallel in our laboratory to support epidemiological investigations, this study specifically focused on phenotypic identification methods, namely, MALDI-TOF MS and VITEK2, that are routinely applicable to live bacterial isolates in time-sensitive diagnostic settings. Furthermore, studies have demonstrated that sporulation significantly alters the metabolic profile of *B. cereus*, reducing the reliability of biochemical identification methods [17,18].

Matrix-assisted laser desorption/ionization time-of-flight mass spectrometry (MALDI-TOF MS) has emerged as a promising technology for bacterial identification, offering advantages in speed, simplicity, and cost-effectiveness. This technique generates unique protein profile “fingerprints” that can be matched against reference databases for rapid identification [15]. In the MALDI-TOF process, microbial proteins are co-crystallized with an energy-absorbing matrix and subjected to pulsed laser irradiation. The matrix facilitates the desorption and ionization of the proteins, which are then accelerated in an electric field within a vacuum tube. The time each ion takes to reach the detector is inversely related to its mass-to-charge ratio (*m*/*z*), allowing the instrument to generate a mass spectrum that reflects the protein composition of the sample [19,20]. This spectral fingerprint serves as a reliable basis for species-level identification and is particularly effective for routine diagnostics. However, for spore-forming bacteria like *B. cereus*, protein composition changes dramatically during sporulation, potentially reducing spectral clarity and identification accuracy [8,21].

In this study, we investigated the relationship among cultivation time, sporulation progression, and MALDI-TOF MS identification accuracy for *Bacillus cereus*. By systematically analyzing identification efficacy at different cultivation stages and directly correlating these with sporulation rates, we aimed to establish optimal processing parameters that maximize identification accuracy while minimizing total analysis time. Through microscopic monitoring of sporulation and comparative analysis with conventional methods, we sought to develop a practical protocol for reliable *B. cereus* identification, potentially reducing total detection time by more than 50% compared to conventional approaches.

## 2. Results

### 2.1. List of Selected Bacillus cereus Isolates and Results of Toxin Gene Testing

Among the thirty *B. cereus* strains analyzed in this study, twenty-eight were isolated from various food sources, and two were obtained from environmental samples, specifically from a poultry farm (Table 1). All strains were subjected to comprehensive toxin gene profiling for six major virulence-associated genes: hblC (encoding hemolytic enterotoxin), bceT (encoding enterotoxin T), entFM (encoding enterotoxin FM), nheA (encoding non-hemolytic enterotoxin), CytK (encoding cytotoxin K), and CER (encoding the emetic toxin cereulide).

The toxin gene analysis revealed that entFM and nheA were the most frequently detected genes, present in 93.3% (28/30) and 80% (24/30) of the isolates, respectively. The hblC gene was detected in 46.7% (14/30) of the strains, while the CytK gene was found in 40% (12/30) of the isolates. The bceT gene was present in 33.3% (10/30) of the strains. Multiple toxin genes were detected in 66.7% (20/30) of the isolates, with some strains carrying up to four different toxin genes simultaneously (Figure S1).

### 2.2. Validation of MALDI-TOF Reliability Through Comparison with VITEK2

The reliability of MALDI-TOF MS for *B. cereus* identification was evaluated by comparing its performance with VITEK2, a well-established biochemical identification system. Both systems were tested using identical strains at three different incubation time points: 12, 24, and 48 h. VITEK2 demonstrated consistent identification rates of 100% (30/30 strains) across all time points tested (12 h, 24 h, and 48 h) (Table 2).

### 2.3. Temporal Progression of Sporulation During Extended Cultivation of B. cereus

**Figure 1:**
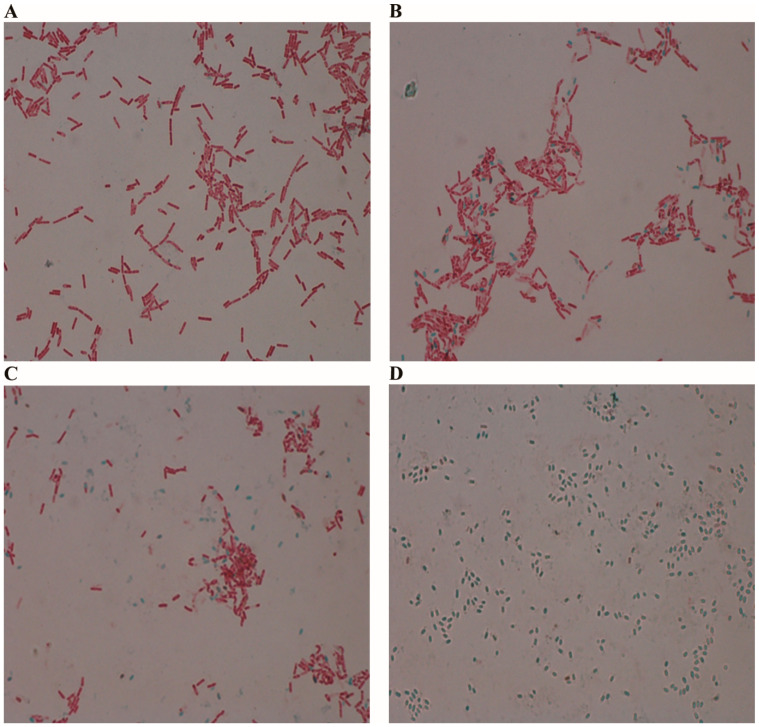
Temporal progression of sporulation in *Bacillus cereus* cultures observed using malachite green stain. (**A**) At 12 h, vegetative cells are stained red with safranin. (**B**) At 24 h, initial spore formation is visible as green structures. (**C**) At 48 h, an increased number of spores with decreasing vegetative cells is observed. (**D**) At 72 h, predominantly mature spores are noted with few remaining vegetative cells. Magnification is ×1000.

**Figure 2:**
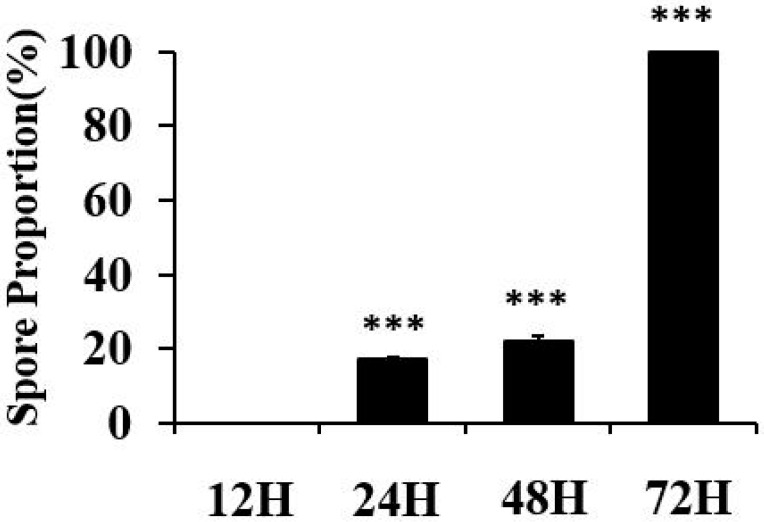
Quantitative analysis of *Bacillus cereus* sporulation over time using malachite green staining. *B. cereus* cultures were incubated for 12, 24, 48, and 72 h. For analysis, bacterial suspensions were standardized to McFarland 0.5 (approximately 1.5 × 10^8^ CFU/mL) before staining with malachite green (spores appear green) and safranin (vegetative cells appear red). For each time point, five independent biological replicates were prepared, with five microscopic fields analyzed per replicate at 1000× magnification. The spore proportion (%) was quantified using ImageJ software by measuring the ratio of the green-stained area (spores) to the total stained area (green + red). Bars represent the mean spore proportion (%) ± standard deviation (*n* = 5 per time point). Statistical analysis was performed using one-way ANOVA (F(3, 16) = 10,676.96, *p* < 0.0001) followed by Tukey’s HSD post-hoc test. Asterisks indicate statistically significant differences compared to the 12-h time point (*** *p* < 0.001).

The declining MALDI-TOF identification rates observed with extended cultivation times prompted an investigation into the potential role of sporulation in this phenomenon. To explore this relationship, we conducted malachite green staining experiments to visualize spore formation across different time points (Figure 1 and Figure 2).

Results from the malachite green staining revealed a clear progression of sporulation that corresponded with the decreased identification rates. At 12 h, cells were exclusively in the vegetative state with no visible spores. However, as cultivation time extended, an increasing proportion of cells exhibited evidence of sporulation, suggesting that spore formation may interfere with the MALDI-TOF identification process.

**Figure 3:**
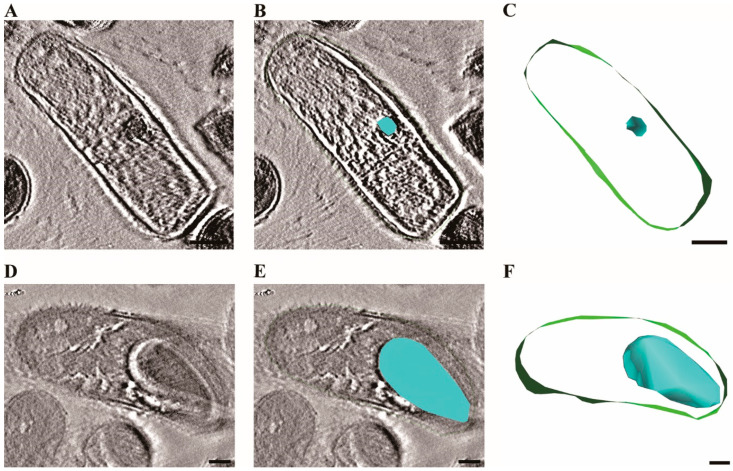
Visualization of intracellular spore formation in *B. cereus* using transmission electron tomography. (**A**) Tomogram of vegetative cell after 12 h of cultivation. (**B**) Tomogram with segmentation highlighting the developing endospore and cell membrane. (**C**) Three-dimensional segmentation model with the endospore noted in blue and cell membrane in green. (**D**) Tomogram of cell after 48 h of cultivation. (**E**) Tomogram with segmentation showing the mature endospore and cell membrane. (**F**) Three-dimensional segmentation was performed using IMOD software, with manual segmentation of cell membranes (green) and developing endospores (blue) based on electron density differences and structural morphology. Scale bars were 200 nm. Quantitative analysis of spore area proportion showed 2.25% in the 12-h sample C compared to 31.46% in the 48-h sample F.

To further characterize the morphological changes associated with sporulation, we employed transmission electron microscopy (TEM) with tomographic analysis (Figure 3).

Tomographic analysis revealed dramatic structural differences between cells cultured for 12 h versus 48 h. In 12-h samples (Figure 3A–C), we observed small, nascent endospores occupying minimal intracellular space, with cellular components remaining largely intact. The three-dimensional reconstruction confirmed the early developmental stage of these spores, appearing as compact structures within the cytoplasm. In contrast, 48-h samples (Figure 3D–F) exhibited mature endospores occupying significant portions of the cellular volume, with pronounced structural differentiation and defined cortex layers. The segmentation models clearly demonstrated the volumetric expansion of spores over time, with mature spores occupying approximately 60% of the intracellular space at 48 h compared to less than 10% at 12 h. These structural transformations coincided precisely with the observed decline in MALDI-TOF identification efficiency, suggesting that the proteomic profiles essential for accurate identification become increasingly altered as cells progress through the sporulation cycle.

**Figure 4:**
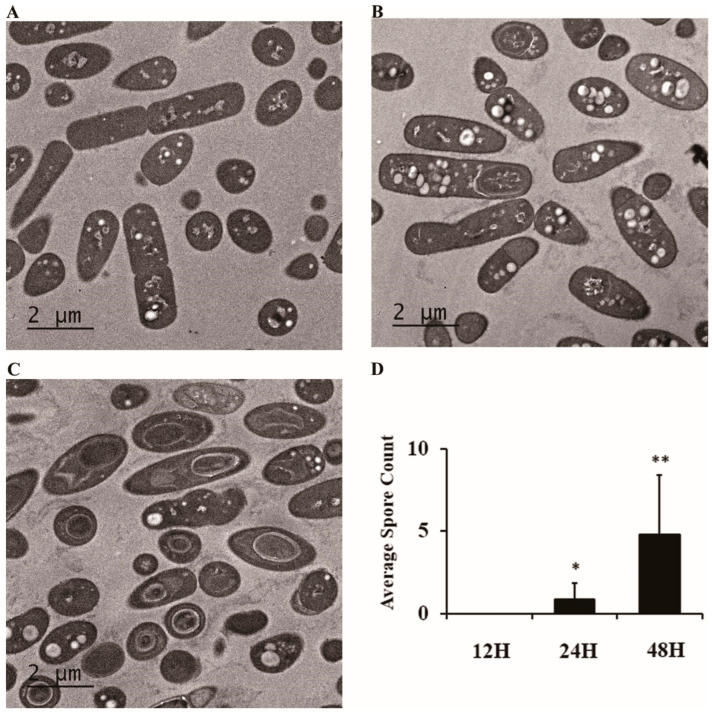
Quantitative analysis of *B. cereus* sporulation progression using transmission electron microscopy. Representative TEM micrographs show the progression of sporulation: (**A**) At 12 h, predominantly vegetative cells with no visible spores are observed. (**B**) At 24 h, initial stages of spore formation are visible within some cells. (**C**) At 48 h, a significant increase in mature spore structures within multiple cells is evident. (**D**) Quantitative analysis of the average spore count per microscopic field at the different incubation times (12 h, 24 h, 48 h). Bars represent the mean spore count ± standard deviation (n = 10 fields analyzed per group). Spore counts increased from an average of 0 at 12 h to 0.9 at 24 h and 4.8 at 48 h. Statistical comparisons were performed using independent t-tests comparing the 24 h and 48 h time points against the 12 h control group. Asterisks indicate statistically significant differences compared to the 12-h time point (* *p* < 0.05, ** *p* < 0.01). Overall significant differences among the three time points were also confirmed using one-way ANOVA followed by Tukey’s HSD post-hoc test.

To quantitatively assess the progression of sporulation, we performed systematic TEM analysis of *B. cereus* cultures at different incubation times (Figure 4).

TEM micrographs revealed distinct morphological differences between samples collected at 12, 24, and 48 h. At 12 h (Figure 4A), cells exhibited typical rod-shaped morphology with homogeneous cytoplasmic content and no evidence of spore formation. By 24 h (Figure 4B), early-stage endospores became visible within approximately 20% of cells, appearing as distinct structures within the cytoplasm with altered electron density. The 48-h samples (Figure 4C) showed dramatic progression of sporulation, with well-defined spore structures apparent in multiple cells.

Quantitative analysis confirmed these observations, with the average number of spores per microscopic field increasing from 0 at 12 h to 0.9 at 24 h and further rising to 4.8 at 48 h (Figure 4D). This increase was statistically significant (*p* < 0.001) and demonstrated a strong temporal correlation with the observed decline in MALDI-TOF identification efficiency. The absence of spores at 12 h corresponded with 100% identification rates, while the presence of spores at 24 and 48 h coincided with reduced identification success (73.3% and 50%, respectively), suggesting a direct relationship between sporulation progression and MALDI-TOF analytical performance.

### 2.4. MALDI-TOF Identification Efficiency over Time and Optimization of the Identification Protocol

**Figure 5:**
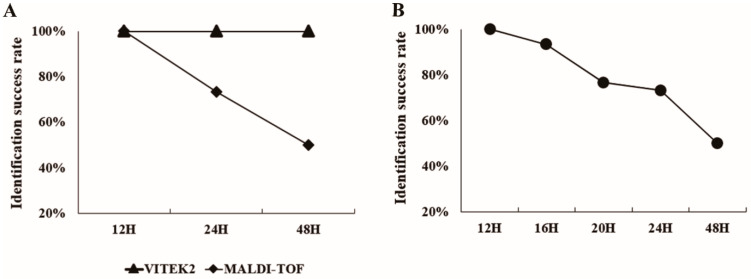
Comparative analysis of *B. cereus* identification efficiency and optimization of incubation time. (**A**) Comparison of identification success rates between VITEK2 and MALDI-TOF MS at three cultivation time points. VITEK2 maintained consistent 100% identification across all time points, while MALDI-TOF MS showed progressive decline from 100% at 12 h to 73.3% at 24 h and 50% at 48 h. (**B**) Detailed analysis of MALDI-TOF MS identification rates at 4-h intervals revealing the optimal time window for accurate identification. High success rates were maintained through 16 h (93.3%), followed by significant declines at 20 h (76.3%), 24 h (73.3%), and 48 h (50%).

To establish the optimal time window for accurate *B. cereus* identification using MALDI-TOF MS and to understand the correlation between sporulation progression and identification efficiency, we conducted a comprehensive comparative analysis of identification methods and performed time interval testing (Figure 5).

While our initial comparison demonstrated equivalent identification accuracy (100%) between VITEK2 and MALDI-TOF MS at 12 h of cultivation (Figure 5A), the two methods diverged significantly with extended incubation times. VITEK2 maintained consistent 100% identification rates across all time points tested, whereas MALDI-TOF MS showed a time-dependent decrease in accuracy, dropping to 73.3% at 24 h and further declining to 50% at 48 h.

To precisely determine the critical time threshold for optimal MALDI-TOF MS performance, we conducted a more detailed analysis at 4-h intervals between 12 and 48 h (Figure 5B). This fine-grained temporal analysis revealed that high identification rates were maintained through 16 h of cultivation (93.3%), after which a significant decline began. By 20 h, the identification rate had decreased to 76.3%, followed by further reductions to 73.3% at 24 h and 50% at 48 h.

The temporal pattern of declining identification rates closely mirrored the progression of sporulation observed in our microscopic and TEM analyses. The onset of significant identification rate decrease (after 16 h) coincided precisely with the beginning of detectable spore formation, confirming our hypothesis that sporulation interferes with MALDI-TOF MS identification accuracy. These findings establish a critical time window of 12–16 h as optimal for *B. cereus* identification using MALDI-TOF MS, providing both high accuracy and significant time savings compared to conventional biochemical methods.

## 3. Discussion

This study demonstrates that optimizing cultivation time is critical for accurate identification of *B. cereus* using MALDI-TOF MS. By systematically analyzing the relationship between sporulation progression and identification accuracy, we have established a practical protocol that significantly reduces detection time while maintaining reliability.

The relationship between sporulation and MALDI-TOF identification accuracy represents the central finding of our research. Our microscopic and TEM analyses clearly demonstrated that sporulation begins after 12 h of incubation, with spore counts increasing from 0 at 12 h to 0.9 at 24 h and 4.8 at 48 h. This progression inversely correlated with MALDI-TOF identification rates, which declined from 100% at 12 h to 73.3% at 24 h and 50% at 48 h. The temporal relationship between these parameters strongly suggests that protein profile alterations during sporulation directly interfere with mass spectral patterns essential for accurate identification (Figure S2).

This difference likely arises from the methods’ distinct principles. MALDI-TOF MS identifies bacteria based on protein profiles, mainly ribosomal proteins. Sporulation significantly alters these profiles, reducing matches to vegetative cell reference spectra (Figure S3, Table S1). Conversely, VITEK2 uses biochemical tests assessing metabolic functions. These broader metabolic characteristics may be less impacted by the specific proteomic shifts affecting MALDI-TOF, allowing VITEK2 to maintain consistent identification performance even as spores develop.

By analyzing identification rates at 4-h intervals, we identified a critical window between 12 and 16 h of cultivation during which MALDI-TOF MS maintains high accuracy (93.3–100%) before dropping significantly after 16 h. Our focus on these earlier time points (12–16 h), despite some standard recommendations for longer incubations, was guided by both international diagnostic standards and practical feasibility considerations. Regulatory protocols such as the Korean Food Code [22], U.S. FDA BAM (Chapter 14) [23], and EU Regulation (EC) No. 2073/2005 [24] recommend a 24-h incubation for *B. cereus* detection in food, reflecting globally harmonized practices aligned with ISO/IEC 17025 [25]. However, our preliminary data indicated that MALDI-TOF MS identification accuracy decreased markedly at 24 h, likely due to sporulation-related alterations in protein profiles. Therefore, investigating these earlier time points (12–16 h) was crucial, as they yielded higher-quality spectra while maintaining compatibility with diagnostic timelines. Although shorter intervals (6–10 h) were initially considered, colonies were not macroscopically visible and thus unsuitable for direct MALDI analysis at those stages. Conversely, the extended time points (24–72 h) were included not for diagnostic applicability, but to allow clear observation of sporulation onset and progression, enabling comparison of spectral quality in relation to sporulation and providing insight into the proteomic changes that compromise identification accuracy.

This finding has immediate practical implications, as it establishes a specific cultivation timeframe that optimizes *B. cereus* identification. Furthermore, this optimization provides significant time advantages compared to conventional methods. While VITEK2 also achieved 100% identification accuracy, it required an additional 16 h of analysis after the initial cultivation, resulting in a total identification time of approximately 28 h. In contrast, our optimized MALDI-TOF protocol can complete the entire process within 12–16 h, offering a substantial time reduction that could be crucial in food safety scenarios requiring rapid response.

The time efficiency of our optimized protocol becomes particularly relevant when considering the virulence potential observed among our isolates. The high prevalence of multiple toxin genes (66.7% of isolates), especially entFM (93.3%) and nheA (80%), underscores the public health risks associated with *B. cereus* contamination. Rapid and accurate identification is essential for effective intervention, especially considering that *B. cereus* can produce heat-stable toxins that remain active even after cooking.

While this study focused specifically on *B. cereus*, the methodology and findings may have broader implications for other spore-forming bacteria. The demonstrated relationship between sporulation and MALDI-TOF identification suggests that similar optimization might be beneficial for other Bacillus species and potentially other spore-forming genera. Further research could explore whether the specific 12–16 h window applies to other spore-formers or if different species require unique optimization parameters.

Future studies could also investigate the specific protein profile changes that occur during sporulation and how these alterations affect MALDI-TOF spectral patterns. Understanding these molecular mechanisms could potentially lead to modified protein extraction protocols or refined identification algorithms that maintain accuracy even in samples with higher spore prevalence. Additionally, exploring cultivation condition modifications that delay sporulation might further extend the window of optimal identification.

In conclusion, this study establishes a practical protocol for optimizing MALDI-TOF MS identification of *B. cereus* by targeting the 12–16 h cultivation window before significant sporulation occurs. This approach maintains 100–93.3% identification accuracy while reducing total analysis time to approximately half that required for conventional biochemical methods. These findings represent a meaningful advancement in food safety diagnostics, offering laboratories a more efficient protocol for identifying this significant foodborne pathogen.

## 4. Materials and Methods

### 4.1. Bacillus cereus Strains and Culture Conditions

A total of 30 *Bacillus cereus* strains were utilized in this study. Twenty-eight strains were isolated from various food samples, including vegetables, chicken, beef, and pork, while the remaining two originated from environmental samples collected at a poultry farm. All strains were isolated and identified by the Gangwon-do Institute of Health and Environment, and a full list of the strains and their sources is presented in Table 1.

To isolate *Bacillus cereus* from food and environmental samples, 25 g of each sample was enriched in 250 mL of tryptic soy broth (TSB; Merck, Darmstadt, Germany) and incubated at 36 °C for 24 h, in accordance with the Korean Food Code, which follows the ISO/IEC 17025 standard for microbial testing. After enrichment, a small aliquot of the culture was collected using a sterile inoculation loop and streaked onto Mannitol Egg Yolk Polymyxin (MYP) agar (Oxoid, Hampshire, UK) using the standard three-phase streaking technique. The plates were then incubated at 30 °C for an additional 24 h. All incubations were performed in a constant-temperature incubator (Sanyo, Osaka, Japan) that undergoes annual temperature calibration and performance validation to ensure operational reliability.

Colonies exhibiting typical *B. cereus* morphology pink coloration with lecithinase-positive halos were selectively collected. To confirm species identity, isolated colonies were subjected to biochemical identification using the VITEK^®^ system (bioMérieux, Marcy-l’Étoile, France) and mass spectrometric identification using the MALDI Biotyper^®^ system (Bruker Daltonics, Bremen, Germany), along with toxin gene profiling. Colonies were considered *B. cereus* only when the MALDI-TOF MS score was ≥2.0 and the VITEK^®^ identification probability was ≥85%.

Confirmed isolates were preserved using the BactoBank™ Microorganism Preservation System (Pulse Scientific Inc., Burlington, ON, Canada) and stored at –25 °C until further use. Prior to experimentation, strains were revived by streaking onto fresh MYP agar and incubating at 30 °C for 24 h to obtain isolated colonies. Single colonies were then sub-cultured twice to ensure purity.

For incubation time analysis, purified *B. cereus* isolates were again inoculated onto MYP agar and incubated at 30 °C. Colonies were harvested at 12, 24, 48, and 72 h post-inoculation for downstream experiments.

### 4.2. Bacillus cereus Toxin Analysis

To confirm the presence of *Bacillus cereus* toxin genes, a pure, isolated single colony was suspended in 100 μL of sterilized distilled water in a 1.5 mL Eppendorf tube. The suspension was boiled at 100 °C for 5 min to lyse the cells and release genomic DNA. After boiling, the sample was immediately centrifuged at 14,000 rpm for 5 min at 4 °C. The supernatant was collected and used as the template DNA for PCR.

Detection of five enterotoxin genes (*hblC*, *bceT*, *entFM*, *nheA*, *CytK*) and the emetic toxin gene (*CER*) was carried out using the PowerCheck™ *Bacillus cereus* 6-toxin Detection Kit (Kogenebiotech, Seoul, Republic of Korea), following the manufacturer’s instructions, including primer sequences and expected product sizes.

PCR amplification was performed using a C1000 Thermal Cycler (Bio-Rad, Hercules, CA, USA) under the following conditions: initial denaturation at 95 °C for 10 min; followed by 35 cycles of denaturation at 95 °C for 30 s, annealing at 60 °C for 30 s, and extension at 72 °C for 30 s; with a final extension at 72 °C for 10 min. The PCR mixture had a total volume of 20 μL and consisted of 5 μL of primer mix, 10 μL of PCR premix, and 5 μL of template DNA. A no-template control (NTC) was included in each PCR run to monitor for potential contamination.

The amplified PCR products were analyzed by electrophoresis on a 1.5% agarose gel prepared with TBE buffer. Electrophoresis was conducted at 120 V for 30 min, and the gel was stained with SHINE nucleic acid dye and visualized under UV illumination using a gel documentation system. A 100 bp DNA ladder (Bioneer, Daejeon, Republic of Korea) was used as a molecular size marker.

### 4.3. Endospore Staining and Counting

*Bacillus cereus* was incubated for 12, 24, 48, and 72 h on MYP agar at 30 °C. At each time point, a sterilized cotton swab was used to collect samples. Each swab was suspended in 3 mL of sterile saline and adjusted to a McFarland standard of 0.5 using the DensiChek Plus Biological Density Meter (bioMérieux, Marcy-l’Étoile, France) in order to ensure a consistent bacterial concentration across all samples. This standardization was conducted solely for the purpose of equalizing the concentration of *B. cereus* cells and not for quantitative cell counting.

A 50 µL aliquot of the adjusted suspension was accurately dispensed onto the Petroff-Hausser Counting Chamber (Hausser Scientific Co., Horsham, PA, USA) using a micropipette. The sample was then heat-fixed using an alcohol lamp. Subsequently, 200 µL of 5% malachite green solution was applied onto the sample area, and the slide was steamed for 5 min to facilitate dye penetration. After cooling, the slide was washed thoroughly with sterile water, counterstained with safranin for 30 s, and rinsed again with sterile water.

After air-drying, the slide was observed under an ECLIPSE NI-U microscope (Nikon Corporation, Tokyo, Japan) at 1000× magnification. Endospores appeared green due to malachite green retention, while vegetative cells were stained red by safranin, allowing for clear differentiation between cell types [26].

The proportion of spores was quantitatively measured using ImageJ software version 1.54 g (National Institutes of Health, Bethesda, MD, USA). Green-stained endospores and red-stained vegetative cells were identified in representative microscopic images, and the area of each was measured separately. The spore fraction was calculated using the formula: green/(green + red), where “green” corresponds to malachite green-stained spores and “red” to safranin-stained vegetative cells.

### 4.4. VITEK2 Microbial Identification

Biochemical identification of the bacterial strains was performed using the VITEK^®^ 2 Compact system (bioMérieux, Marcy-l’Étoile, France). A colony exhibiting a pink color on Mannitol Egg Yolk Polymyxin (MYP) agar was subcultured onto nutrient agar (Merck, Darmstadt, Germany). The resulting bacterial suspension was prepared in 3 mL of sterile saline and adjusted to a McFarland standard of 2.0 using the DensiChek Plus Biological Density Meter (bioMérieux, Marcy-l’Étoile, France).

The Bacillus identification card (BCL; bioMérieux, Marcy-l’Étoile, France) was automatically filled in the VITEK vacuum chamber, sealed, and incubated at 35.5 °C. Biochemical reactions were recorded every 15 min for up to a maximum of 16 h. Identification data were analyzed using the VITEK 2 database version 7.01. The identification results were considered valid when the probability was ≥85%.

### 4.5. MALDI-TOF MS Analysis

Matrix-assisted laser desorption/ionization time-of-flight mass spectrometry (MALDI-TOF MS) analysis was performed using the direct transfer method with the MALDI Biotyper^®^ system (Bruker Daltonics, Bremen, Germany). A single colony grown on MYP agar medium at various time points (12, 24, 48, and 72 h) was collected using a sterile loop and directly applied to a designated spot on the MALDI target plate (Bruker Daltonics, Bremen, Germany).

Subsequently, 1 µL of matrix solution consisting of α-cyano-4-hydroxycinnamic acid (Bruker Daltonics, Bremen, Germany) dissolved in an organic solvent (acetonitrile 50%, water 47.5%, and trifluoroacetic acid 2.5%) was added to the spot. The sample was allowed to air-dry at room temperature, during which co-crystallization of the matrix and the microbial sample occurred [27].

Mass spectra were acquired in the mass-to-charge (*m*/*z*) range of 2000 to 20,000 and analyzed using the MALDI Biotyper^®^ software version 3.4 with the Bruker reference library version 4.1 [28]. Identification scores were interpreted as follows: scores ≥ 2.0 were considered reliable species-level identification, scores between 1.7 and 1.9 were considered reliable genus-level identification, and scores < 1.7 were considered unreliable identification. For this study, only identifications with scores ≥ 2.0 were considered positive for *B. cereus* [16]. To ensure consistent comparison between platforms, colonies analyzed using MALDI-TOF MS and VITEK2 were selected from the same agar plate and cultivation time point.

### 4.6. Statistical Analysis

All data are presented as mean ± standard deviation (SD). Statistical analyses were performed using Microsoft Excel 2016 (Microsoft Corp., Redmond, WA, USA). Comparisons between two individual time points (e.g., 12 h vs. 24 h) were conducted using unpaired Student’s t-tests, and statistical significance was determined at a *p*-value of <0.05. To assess overall differences across all incubation time groups (12 h, 24 h, 48 h, and 72 h), one-way analysis of variance (ANOVA) was performed.

When the ANOVA indicated significant differences, Tukey’s honest significant difference (HSD) test was applied manually to identify pairwise group differences. For Tukey HSD, actual *p*-values were interpreted, and significance levels are specified in the figure legends.

### 4.7. Sample Preparation and Transmission Electron Microscopy

For TEM analysis, *B. cereus* cells cultured for 12, 24, and 48 h were harvested by gentle centrifugation at 3000 rpm for 5 min. The cell pellets were washed twice with phosphate-buffered saline (PBS, pH 7.4) and fixed with 2.5% glutaraldehyde in 0.1 M phosphate buffer for 2 h at 4 °C. After fixation, samples were washed three times with the same buffer (10 min each) and post-fixed with 1% osmium tetroxide for 1 h at room temperature. The fixed samples were then dehydrated using a graded ethanol series (50%, 70%, 80%, 90%, and 100%) for 30 min each at 4 °C, with a final overnight incubation in 100% ethanol.

Samples were infiltrated with increasing concentrations of Spurr’s resin (25%, 50%, 75%) in propylene oxide for 2 h each, followed by infiltration with 100% resin overnight. Infiltration and embedding were performed at the Kangwon Center for Systems Imaging. The samples were placed in embedding molds and polymerized at 60 °C for 48 h. Ultrathin sections (approximately 70–80 nm) for conventional TEM and thicker sections (150–200 nm) for electron tomography were prepared using a Leica EM UC7 ultramicrotome equipped with a diamond knife. The sections were collected on 75-mesh copper grids.

For contrast enhancement, grids were stained with 1% uranyl acetate for 20 min in the dark, washed briefly with distilled water, and counterstained with 0.02% lead citrate for 10 min [29]. Micrographs were collected on a Tecnai 10 transmission electron microscope (FEI, Hillsboro, OR, USA) operated at 100 kV and recorded on an UltraScan 1000 CCD camera (Gatan, Pleasanton, CA, USA) at a magnification of 4200×, corresponding to a pixel size of 2.44 nm. For each time point (12, 24, and 48 h), at least 20 fields were randomly selected and imaged to ensure representative sampling [13].

### 4.8. Electron Tomography Analysis

For electron tomography, tilt series were acquired from −60° to +60° with 4° increments using the same microscope settings as described in Section 4.6. The acceleration voltage was maintained at 100 kV during the acquisition of the complete tilt series.

Alignment, 3D reconstruction, visualization, and segmentation of the tomograms were all performed using the IMOD software package version 4.12 (University of Colorado, Boulder, CO, USA) [30]. Standard alignment procedures were applied to the tilt series, followed by 3D reconstruction using the simultaneous iterative reconstruction technique (SIRT) with three iterations to optimize the contrast and resolution of the final tomogram [31]. Cell membranes and developing endospores were manually segmented to generate the 3D models shown in Figure 3, with membranes highlighted in green and endospores in blue.

### 4.9. Quantitative Analysis of Sporulation

Spore counts were determined by analyzing TEM micrographs from each time point (12, 24, and 48 h). A structure was classified as a spore based on the presence of a distinct cortex layer and electron-dense regions within the developing spore. The number of spores per field was counted from at least 20 randomly selected fields, and the average was calculated for each time point.

For time interval testing to determine the optimal window for MALDI-TOF MS identification, *B. cereus* cultures were analyzed at 4-h intervals between 12 and 48 h of incubation. At each time point, both MALDI-TOF MS identification and sporulation rates were assessed using the methods described above.

## Figures and Tables

**Table 1:** Toxin gene profiles of *Bacillus cereus* isolates. Each row presents both the experimental ID (1–30) and the corresponding strain ID issued by the Gangwon-do Institute of Health and Environment.

Experimental ID	Strain ID	Identified Species	Characteristics	Source
1	GWB66191186686	*Bacillus cereus*	becT, entFM	Food
2	GWB66191186689	*Bacillus cereus*	becT, hblC, nheA, entFM	Food
3	GWB66191186690	*Bacillus cereus*	hblC, nheA, entFM	Food
4	GWB66191186691	*Bacillus cereus*	becT, nheA, entFM	Food
5	GWB66191186693	*Bacillus cereus*	nheA, entFM	Food
6	GWB66191186697	*Bacillus cereus*	becT, hblC, nheA, entFM	Food
7	GWB66191186698	*Bacillus cereus*	hblC, nheA, entFM	Food
8	GWB66191186699	*Bacillus cereus*	becT, hblC, entFM	Food
9	GWB66191186700	*Bacillus cereus*	nheA, entFM	Food
10	GWB66191186701	*Bacillus cereus*	becT, hblC, nheA, CytK, entFM	Food
11	GWB66191186702	*Bacillus cereus*	hblC, nheA, CytK, entFM	Food
12	GWB66191186703	*Bacillus cereus*	hblC, nheA, CytK, entFM	Food
13	GWB66191186704	*Bacillus cereus*	becT, hblC, nheA, entFM	Food
14	GWB66191186706	*Bacillus cereus*	becT, hblC, nheA, CytK, entFM	Food
15	GWB66191186707	*Bacillus cereus*	becT, hblC, nheA, CytK, entFM	Food
16	GWB66200697249	*Bacillus cereus*	hblC, entFM	Food
17	GWB66200697252	*Bacillus cereus*	nheA, entFM	Food
18	GWB66200697253	*Bacillus cereus*	hblC, nheA, CytK, entFM	Food
19	GWB66200697254	*Bacillus cereus*	nheA, CytK, entFM	Food
20	GWB66200697302	*Bacillus cereus*	becT, hblC, nheA, CytK, entFM	Food
21	GWB66200697256	*Bacillus cereus*	hblC, nheA, entFM	Food
22	GWB66200697258	*Bacillus cereus*	nheA, CytK, entFM	Food
23	GWB66200697276	*Bacillus cereus*	hbIC, nheA, CytK, entFM	Food
24	GWB66200697277	*Bacillus cereus*	nheA, CytK, entFM	Food
25	GWB66200697278	*Bacillus cereus*	nheA, entFM	Food
26	GWB66200697279	*Bacillus cereus*	becT, hblC, nheA, CytK	Food
27	GWB66200697281	*Bacillus cereus*	nheA, entFM	Food
28	GWB66200697283	*Bacillus cereus*	nheA, entFM	Food
29	GWB66200697289	*Bacillus cereus*	nheA, entFM, CytK	Environment
30	GWB66200697295	*Bacillus cereus*	becT, nheA, entFM, CytK	Environment

**Table 2:** Analysis of identification rate over time.

(Number of Completed Identifications/Number of Strains)			
TIME	12 H	24 H	48 H
VITEK2	100% (30/30)	100% (30/30)	100% (30/30)
MALDI-TOF	100% (30/30)	73.3% (22/30)	50% (15/30)

## Data Availability

The datasets used and/or analyzed during the current study are available from the corresponding author on reasonable request.

## Supplementary Materials

The following supporting information can be downloaded at: https://www.mdpi.com/article/10.3390/ijms26094355/s1.

## Abbreviations

BCL: Bacillus identification card, bceT: Enterotoxin T, CER: Cereulide, CytK: Cytotoxin K, DNA: Deoxyribonucleic acid, entFM: Enterotoxin FM, hblC: Hemolytic enterotoxin C, IMOD: Image processing software for tomography, MALDI-TOF MS: Matrix-assisted laser desorption/ionization time-of-flight mass spectrometry, MYP: Mannitol egg yolk polymyxin, nheA: Non-hemolytic enterotoxin A, NTC: No-template control, PCR: Polymerase chain reaction, PBS: Phosphate-buffered saline, SD: Standard deviation, SIRT: Simultaneous iterative reconstruction technique, TBE: Tris-Borate-EDTA, TEM: Transmission electron microscopy, TSB: Tryptic soy broth, UV: Ultraviolet, VITEK2: Automated biochemical identification system.
