# Supplementary material for: Optimizing MALDI-TOF Mass Spectrometry for the Identification of Bacillus cereus: The Impact of Sporulation and Cultivation Time
Source: Int J Mol Sci. 2025 May 4;26(9):4355. doi: 10.3390/ijms26094355 (PMC12072534; doi:10.3390/ijms26094355)
Supplement: Supplementary file 1 [file ijms-26-04355-s001.zip › ijms-3574164-supplementary.pdf]

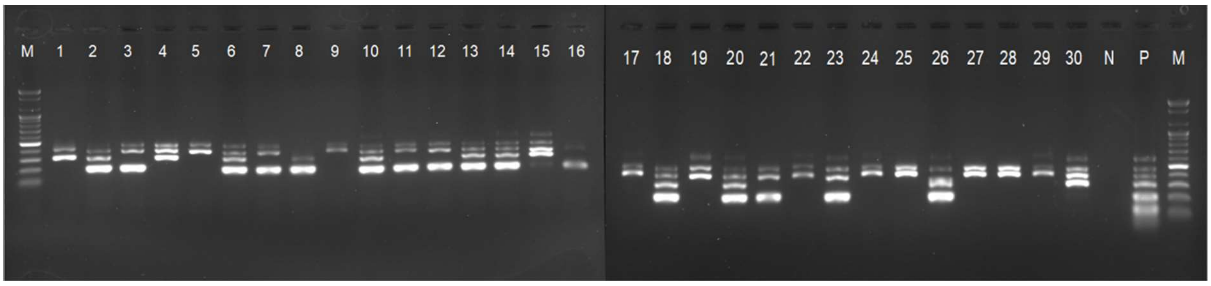

Figure S1. Agarose gel electrophoresis of PCR products amplified from toxin genes in *Bacillus cereus* isolates. PCR amplification of six toxin genes (*hblC*, *bceT*, *entFM*, *nheA*, *CytK*, and *CER*) was performed using the PowerCheck™ *Bacillus cereus* 6-toxin Detection Kit. Amplified products were separated on 1.5% TBE agarose gels and visualized under UV light. Isolate numbers 1–30 correspond to experimental sample IDs, each uniquely matched to an institutional strain ID (e.g., GWB66191186686) as listed in Table 1. M represents 100 bp DNA ladder (Bioneer D-1030), N represents no-template control, and P represents positive control containing all six toxin genes. The expected sizes of the amplified products were: *CytK* (565 bp), *nheA* (499 bp), *entFM* (414 bp), *bceT* (335 bp), *hblC* (241 bp), and *CER* (134 bp). The prevalence rates of the toxin genes among the 30 isolates were: *entFM* (93.3%), *nheA* (80%), *hblC* (46.7%), *CytK* (40%), and *bceT* (33.3%). No isolate was positive for the emetic toxin gene *CER*. Multiple toxin gene patterns were observed in 66.7% of the isolates, confirming the virulence potential of the studied *B. cereus* strains.

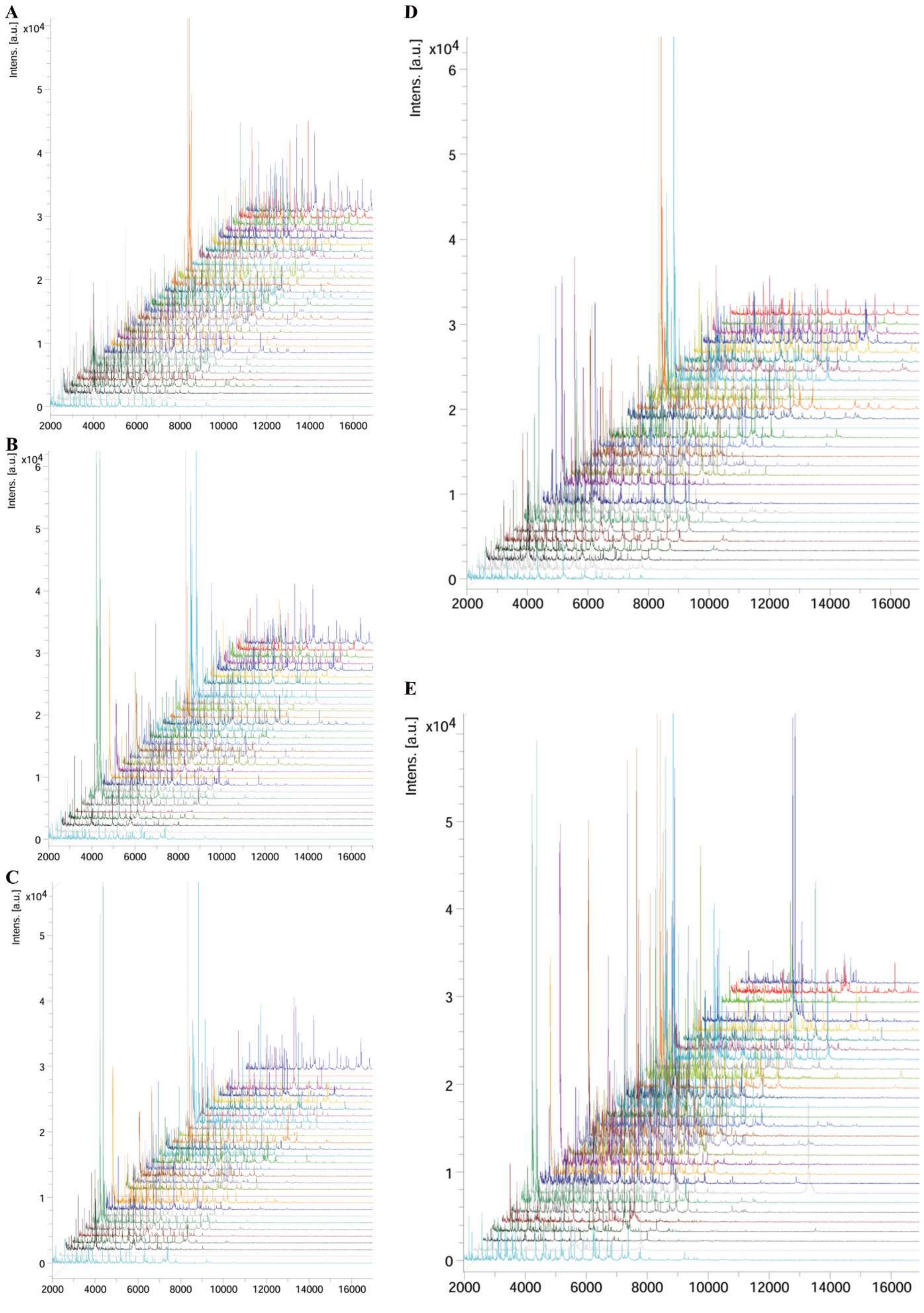

Figure S2. MALDI-TOF MS protein profiles of *Bacillus cereus* at different cultivation times. Representative MALDI-TOF mass spectra showing protein profiles of *B. cereus* cultured for different

time periods: (A) 12 hours, (B) 16 hours, (C) 20 hours, (D) 24 hours, and (E) 48 hours. Mass spectra were acquired in the m/z range of 2,000 to 20,000 Da using the Bruker MALDI Biotyper system. The spectral profiles demonstrate distinct changes in protein composition as cultivation time increases. At 12 and 16 hours, consistent and well-defined peak patterns are observed, corresponding to high identification rates (100% and 93.3%, respectively). As cultivation time extends to 20, 24, and 48 hours, progressive alterations in peak intensity and distribution are evident, correlating with decreased identification accuracy (76.3%, 73.3%, and 50%, respectively). These changes in protein profiles directly correspond to the progression of sporulation observed in microscopic analyses. The characteristic ribosomal protein peaks in the 4,000-10,000 m/z range that are essential for reliable species identification show significant variation with increased cultivation time, highlighting the importance of standardizing cultivation duration for accurate MALDI-TOF MS-based identification of *B. cereus*.

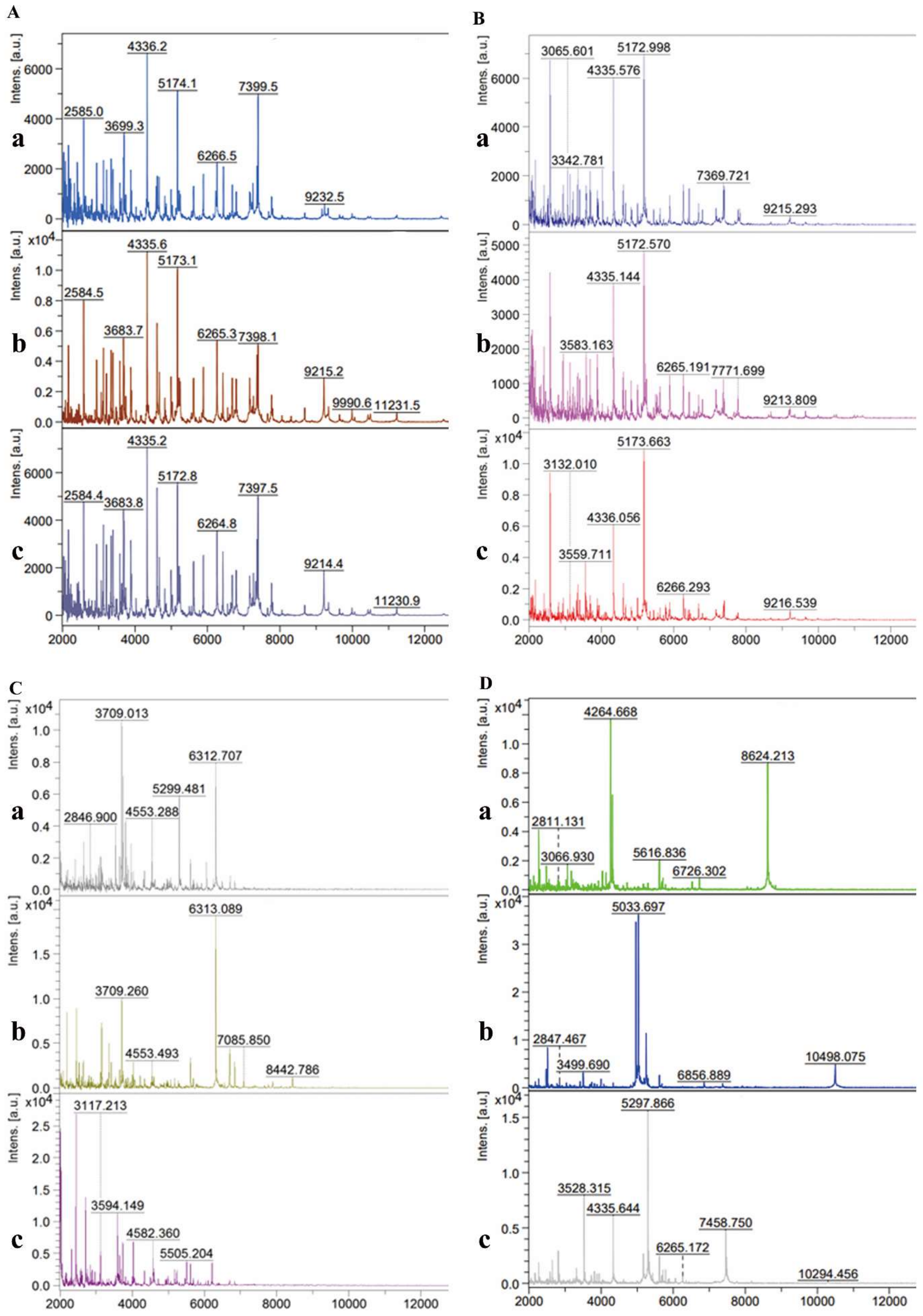

Figure S3. Annotated representative MALDI-TOF mass spectra of *Bacillus cereus* isolates. Spectra were obtained from isolates cultivated at (A) 12 h, (B) 16 h, (C) 24 h, and (D) 48 h. Each panel

contains three representative spectra from distinct isolates, labeled as (a–c), respectively. In panels A and B, consistently observed peaks such as m/z 4335, 5174, and 6265 were frequently detected and are considered putative ribosomal protein markers associated with high-confidence identification. In contrast, panels C and D show low signal intensity and loss of peak clarity, likely due to sporulation-related degradation of protein profiles. These spectral changes were reproducible across replicate isolates, and the spectral consistency observed at 12 h and 16 h suggests their potential utility as stable reference profiles for identification. Further investigation is warranted to better understand the spectral characteristics associated with sporulation stages.

Time	Sub-figure	Experimental ID	Observed Peak (m/z)	Putative Assignment	Confidence
12 h	(a)	8	4335	Putative ribosomal protein marker	High
			5174	Putative ribosomal protein marker	High
			7399	Unknown	High
12 h	(b)	9	4335	Putative ribosomal protein marker	High
			5173	Putative ribosomal protein marker	High
			6265	Putative ribosomal protein marker	High
12 h	(c)	10	4335	Putative ribosomal protein marker	High
			5172	Putative ribosomal protein marker	High
			7398	Unknown	High
16 h	(a)	10	4335	Putative ribosomal protein marker	High
			5172	Putative ribosomal protein marker	High
			7369	Unknown	High
16 h	(b)	11	4335	Putative ribosomal protein marker	High

			5172	Putative ribosomal protein marker	High
			6265	Putative ribosomal protein marker	High
16 h	(c)	19	4336	Putative ribosomal protein marker	High
			5173	Putative ribosomal protein marker	High
			6266	Putative ribosomal protein marker	High
24 h	(a)	3	2847	Unknown	Low
			4553	Unknown	Low
			6312	Unknown	Low
24 h	(b)	11	3709	Unknown	Low
			7085	Unknown	Low
			8443	Unknown	Low
24 h	(c)	12	3594	Unknown	Low
			4582	Unknown	Low
			5505	Unknown	Low
48 h	(a)	8	4264	Unknown	Low
			5616	Unknown	Low
			8624	Unknown	Low
48 h	(b)	11	5033	Unknown	Low
			6856	Unknown	Low
			10498	Unknown	Low
48 h	(c)	13	4335	Putative ribosomal protein marker	Low
			7458	Unknown	Low
			10294	Unknown	Low

Table S1. Observed m/z peaks in representative MALDI-TOF MS spectra of Bacillus cereus isolates. Each sub-figure (a–c) corresponds to spectra shown in Figure S3 panels A–D, representing different cultivation time points. Peaks listed were visually selected based on high signal intensity and reproducibility.

*Note**:** Several peaks (e.g., m/z 4335, 5174, 6265) are commonly reported as ribosomal protein-associated features in Bacillus cereus and Bacillus spp., as suggested in prior MALDI-TOF MS studies (Fenselau and Demirev, 2001; Lasch et al., 2008). Comprehensive assignment of all observed peaks was beyond the scope of this study. Peaks observed primarily in low-confidence spectra (24h, 48h) potentially correspond to spore-specific proteins (e.g., SASPs) or degradation products, but were not explicitly assigned.*
